# Parastomal hernia prevention and repair in Australasia: A binational CSSANZ survey of contemporary practice

**DOI:** 10.1007/s10029-025-03581-8

**Published:** 2026-02-09

**Authors:** Rathin Gosavi, Paul McMurrick, Thang Chien Nguyen, Vignesh Narasimhan

**Affiliations:** 1 Department of Colorectal Surgery, Cabrini Health, Melbourne, VIC Australia; 2 Department of Colorectal Surgery, Monash Health, Melbourne, VIC Australia; 3 Department of Surgery (School of Clinical Sciences at Monash Health), Monash University, Melbourne, VIC Australia

**Keywords:** Stoma creation, Parastomal hernia, Parastomal hernia repair, Prophylactic mesh

## Abstract

**Background:**

Parastomal hernia (PSH) is a debilitating long-term complication of stoma formation, often required as part of curative or palliative treatment for colorectal cancer. As a common downstream consequence of cancer surgery, PSH contributes significantly to chronic morbidity and impairs quality of life, yet practices surrounding its prevention and repair remain heterogeneous. Despite randomised evidence supporting prophylactic mesh, adoption is limited, and operative approaches to elective and emergency repair vary widely.

**Methods:**

A cross-sectional survey of colorectal surgeons in Australia and New Zealand was conducted via the Colorectal Surgical Society of Australia and New Zealand (CSSANZ) to assess current PSH management. The survey examined stoma creation practices, prophylactic and therapeutic mesh use, operative techniques, and responses to clinical vignettes. Subgroup analyses explored variation by surgeon seniority, practice setting, and country.

**Results:**

Ninety-three surgeons responded (93/365, 25.5%), including 74/93 (79.6%) from Australia and 19/93 (20.4%) from New Zealand; 79/93 (84.9%) practised in metropolitan centres and 39/93 (41.9%) had > 15 years’ experience. Routine prophylactic mesh use at stoma formation was reported by 11/93 (11.8%). For elective repair (n = 89), open access was preferred by 48/89 (53.9%) and Sugarbaker repair was the most common configuration (40/89, 44.9%). Technique selection differed by access: among surgeons favouring minimally invasive surgery (n = 41), 33/41 (80.5%) selected Sugarbaker, whereas those favouring open surgery (n = 48) more often selected keyhole (22/48, 45.8%) or retrorectus “sandwich” repair (18/48, 37.5%) (p < 0.00001). In the emergency small-bowel obstruction vignette, surgeons with > 15 years’ experience more often favoured mesh use than those with ≤ 15 years (21/39, 53.8% vs 10/50, 20.0%; p = 0.0015). Overall operative volume was low, with 65/93 (69.9%) reporting 0–5 PSH repairs per year.

**Conclusions:**

PSH prevention and repair across Australasia is marked by wide variability, low uptake of prophylactic mesh, and inconsistent technique selection. Operative approach strongly influenced repair configuration, and seniority appeared to drive emergency decision-making. Addressing PSH represents an important opportunity to reduce treatment-related morbidity in patients with pelvic malignancies undergoing stoma formation.

## Introduction

Parastomal hernia (PSH) is one of the most frequent long-term complications following stoma formation [1], with incidence estimates ranging from 30 to 50% [2] depending on follow-up duration and detection modality [3]. Clinically significant PSH can result in pain, cosmetic deformity, appliance dysfunction, obstruction, and the need for reoperation [4, 5]. Despite its prevalence and morbidity, wide variation persists in both preventive and therapeutic strategies across surgical units [1].

Prophylactic mesh placement at the time of index stoma formation has been shown in randomised trials to reduce PSH rates without significantly increasing complications such as infection or fistula [6–8]. However, uptake into routine practice remains low, potentially due to concerns regarding technical complexity, mesh type, plane of placement, and long-term safety [9]. Similarly, operative repair of PSH is technically challenging, with a range of options including onlay, sublay, and intraperitoneal approaches, with or without mesh, and with highly variable recurrence rates [10, 11].

The optimal prevention and management strategy for PSH remains elusive, in part due to the lack of consensus guidelines and the absence of large-scale prospective studies in routine practice [1, 9]. Capturing contemporary variation in surgical opinion and practice is an important step in informing research priorities, clinical trials design, and eventual consensus-building efforts.

This study aimed to characterise current practice patterns among colorectal surgeons in Australia and New Zealand regarding PSH prevention and repair. Through a binational survey conducted via the Colorectal Society of Australia and New Zealand (CSSANZ), we sought to capture preferences in index stoma formation, prophylactic mesh use, repair techniques, and attitudes towards emerging technologies and evidence gaps.

## Methods

### Study design and participants

We undertook a cross-sectional, web-based survey of practising colorectal surgeons and current colorectal fellows listed on the CSSANZ membership roll. All practising members were eligible; retired, non-practising or non-colorectal affiliate members were not the target population. The questionnaire was sent to the CSSANZ mailing list on 29 August 2025, with a single reminder on 12 September 2025, and the survey closed on 29 September 2025. This was therefore a single-stage census of the available membership rather than a sampled survey.

### Survey development

The instrument was developed by a multidisciplinary group (colorectal surgery, abdominal wall/hernia, surgical education) after a focused literature review on parastomal hernia prevention and repair. Items were organised into four sections: (1) respondent characteristics and operative volume; (2) index stoma formation and prophylactic mesh; (3) elective PSH repair (approach, configuration, mesh type); and (4) clinical vignettes exploring emergency and redo scenarios. Vignettes asked respondents to indicate their preferred management assuming they were the responsible operating surgeon or supervising consultant for the case. Question types included fixed-choice responses and 5-point Likert items (very unlikely to very likely).

Drafts were pilot tested twice with five CSSANZ colorectal surgeons (public–private mix; Australia and New Zealand) using cognitive debriefing to assess clarity, content coverage and skip logic. Minor wording changes were made, but no items were removed. No previously validated external scales were used because no instrument specific to PSH practice was available. The final questionnaire is provided in Supplement 1.

### Survey administration, anonymity and ethics

The survey was administered in Qualtrics using unique links to reduce duplicate entries; device-level protections within the platform further limited multiple submissions. Data were stored on secure Monash Health servers with access restricted to the study team. Ethics approval was obtained from Monash Health (RES-25–0000-503Q). Participation was voluntary and anonymous, with electronic informed consent required at survey entry; no patient-level data were collected.

### Data management and statistical analysis

Responses were exported from Qualtrics and analysed in Microsoft Excel and Python (v3.10). We reported counts and percentages for all categorical variables. For Likert-scale items we present the full distribution in figures; for the clinical vignettes we also report collapsed categories (likely/very likely vs unlikely/very unlikely), with neutral shown separately, because this reflected how surgeons make decisions in practice. No data imputation was undertaken; analyses used available cases and item-level denominators are reported where they differ from the total sample.

Prespecified subgroup comparisons were: country (Australia vs New Zealand), practice location (metropolitan vs provincial), and surgeon seniority (fellow, < 5 years, 5–15 years, > 15 years). Group differences were assessed using Pearson’s chi-squared test; Fisher’s exact test was used when expected cell counts were < 5. Two-sided p < 0.05 was considered statistically significant. Selected effects are presented as odds ratios with 95% confidence intervals. Given the descriptive intent of the survey and the limited sample size, no weighting, no adjustment for non-response, and no correction for multiple comparisons were applied; findings from subgroup analyses should therefore be interpreted as exploratory.

## Results

### Demographics

Ninety-three colorectal surgeons responded (93/365; 25.5%). Most respondents practised in Australia (80%, 74/93), with the remainder from New Zealand (20%, 19/93). The majority worked in metropolitan settings (85%, 79/93), and seniority was skewed toward experienced consultants: 42% (39/93) had > 15 years’ experience, 29% (27/93) had 5–15 years, 17% (16/93) had < 5 years, and 12% (11/93) were current fellows. Annual parastomal hernia repair volume was low overall, with 70% (65/93) performing 0–5 repairs per year. Further demographic detail is provided in Table 1.

**Table 1:** Participant demographics

Domain	Category	N (%)
Country	Australia New Zealand	74 (80) 19 (20)
Practice location	Metropolitan Provincial	79 (85) 14 (15)
Seniority	Fellow Consultant < 5 years Consultant 5–15 years Consultant > 15 years	11 (12) 16 (17) 27 (29) 39 (42)
Annual PSH repairs	0–5 5–10 > 10	65 (70) 27 (29) 1 (1)

Abbreviations: PSH, parastomal hernia; NZ, New Zealand.

### Stoma formation techniques

**Fig. 1:**
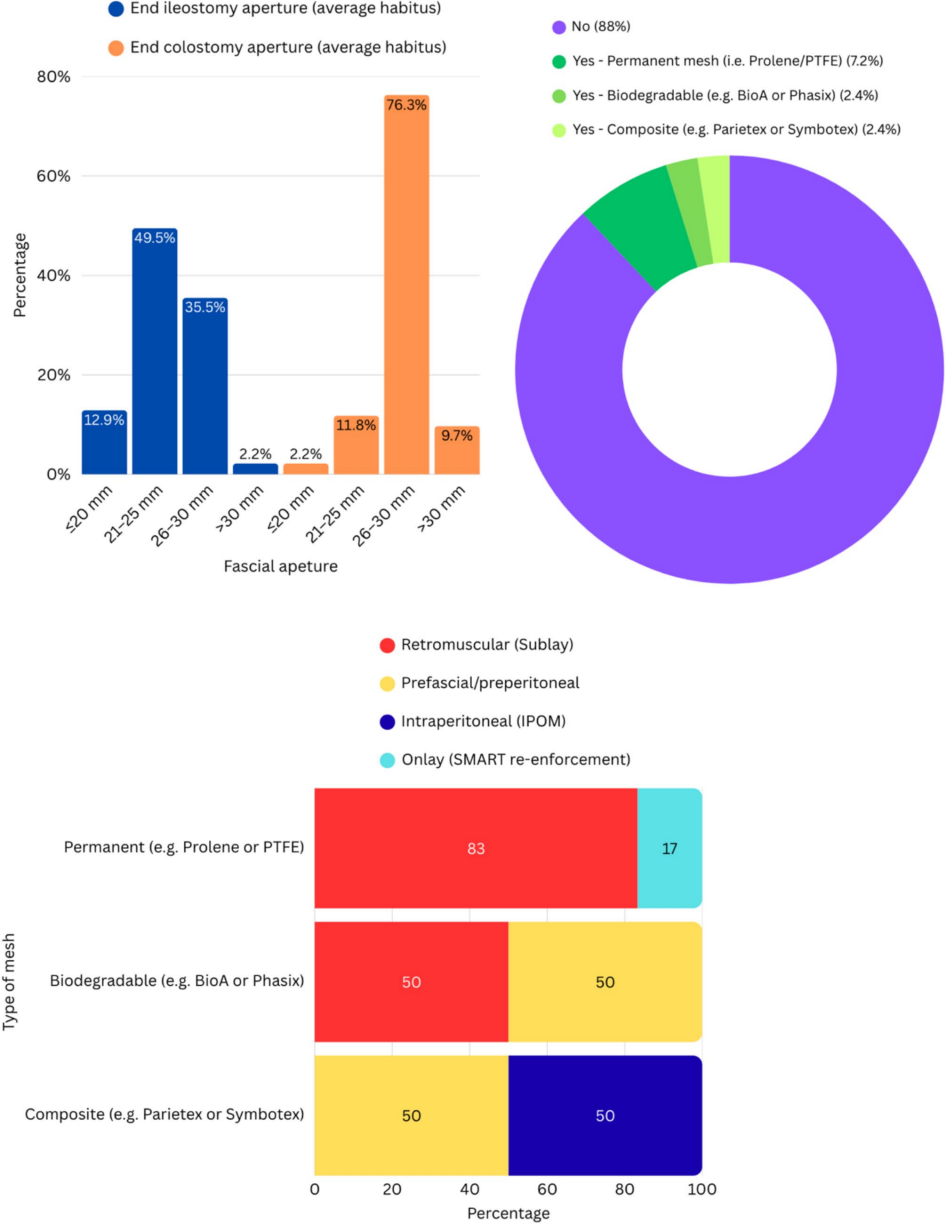
Stoma creation practices and prophylactic mesh use. A) Preferred fascial aperture size for end ileostomy and end colostomy in patients with average habitus (n = 93). B) Use of prophylactic mesh at the time of stoma creation and, among users, the distribution of mesh materials (overall n = 93; mesh users n = 10). C) Placement plane stratified by mesh type among prophylactic mesh users (row-percent within each mesh type; n = 10). IPOM = intraperitoneal onlay mesh; sublay = retromuscular

The majority of respondents reported using a linear vertical fascial incision for stoma creation (69/93, 74%), with fewer preferring cruciate (25%) or circular (1%) incisions. As shown in Fig. 1 A, a 21–25 mm fascial aperture was the most commonly selected size for end ileostomy, whereas 26–30 mm was preferred for end colostomy. Routine use of prophylactic mesh at the time of stoma formation was infrequent, reported by only 11/93 surgeons (12%) (Fig. 1B). Among mesh users, permanent synthetic materials were most commonly employed (6/10), and the favoured placement plane was retromuscular (6/10) (Fig. 1C).

### Mesh in elective PSH repair

**Fig. 2:**
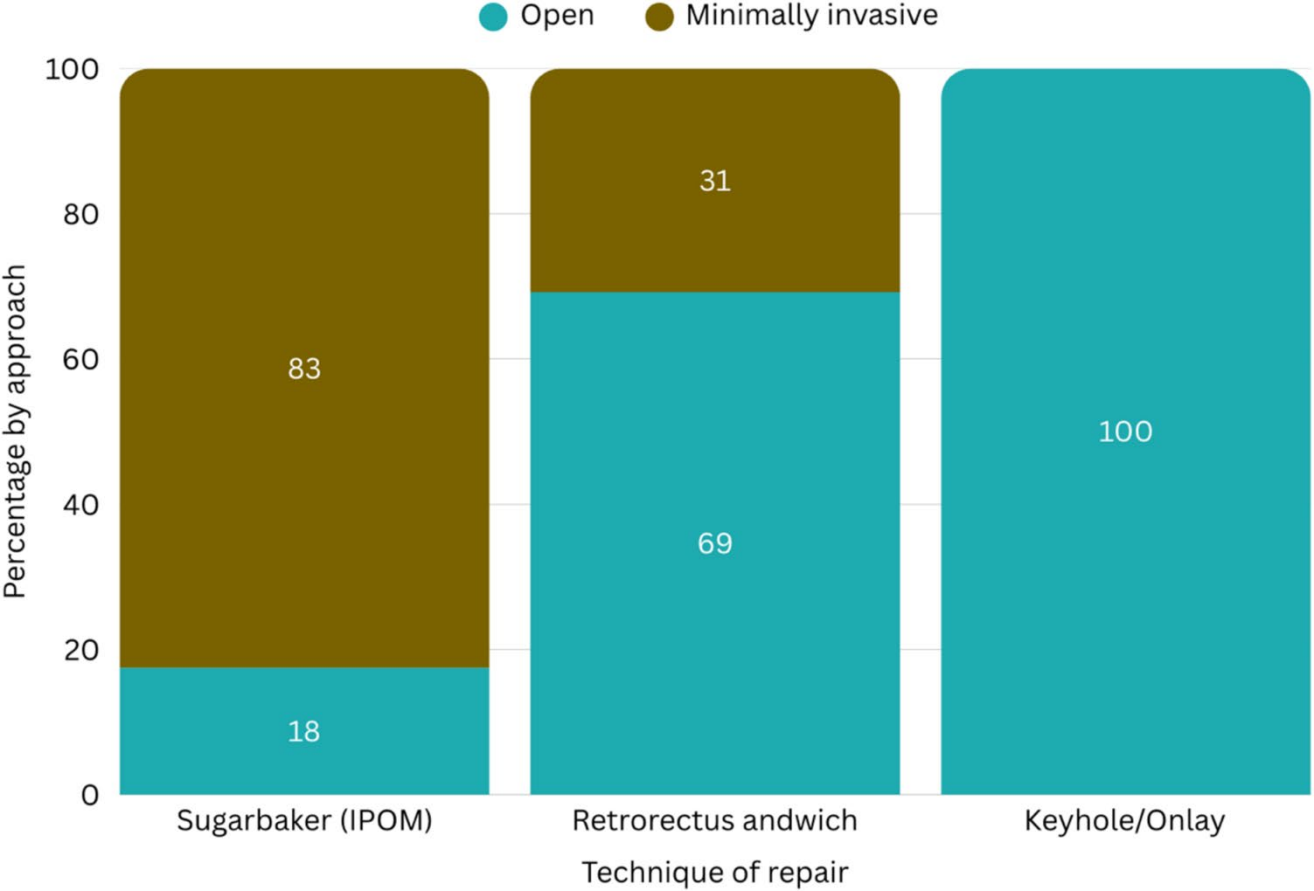
Operative approach by elective parastomal hernia repair technique. Bars show the within-technique proportion performed open vs minimally invasive. Sample sizes: Sugarbaker (IPOM) n = 40, retrorectus “sandwich” n = 26, keyhole/onlay n = 22, no mesh n = 1. The no-mesh category represents a single open case

For elective repair, open access was preferred in 48/89 (54%), with the remainder favouring a minimally invasive approach. Sugarbaker (intraperitoneal onlay mesh) repair (40/89, 45%) was the most common approach to elective repair. Only one respondent (1%) reported routinely performing elective repair without mesh. Among 82 respondents who disclosed mesh type during elective repair, composite meshes were used by 32/82 (39%), permanent meshes by 31/82 (38%), and biodegradable meshes by 19/82 (23%) (Fig. 2).

### Elective case scenario

**Fig. 3:**
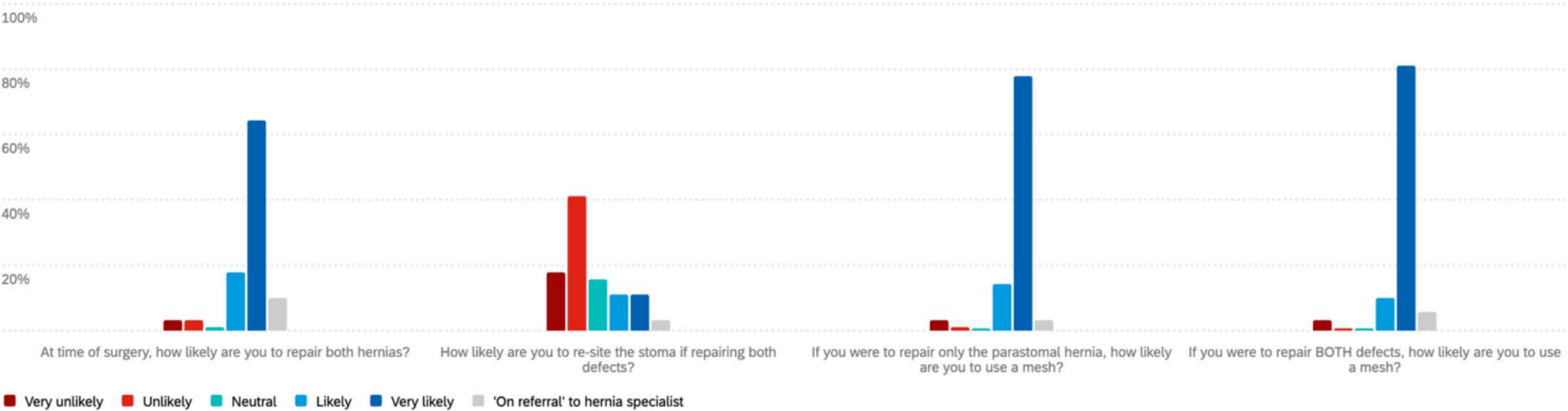
Elective scenario of recurrent parastomal hernia with concurrent ventral hernia

In a case vignette involving a symptomatic, non-obstructed recurrent parastomal hernia with concurrent midline incisional hernia (Fig. 3), the majority of respondents (73/89, 82.0%) opted to repair both defects. Mesh use was nearly universal in both contexts: 91.0% (81/89) would use mesh when repairing both, and 92.1% (82/89) for parastomal repair alone. Re-siting the stoma was uncommon, with only 22.5% (20/89) likely or very likely to do so, and 59.5% (53/89) unlikely or very unlikely. Among those electing mesh repair, preferences were evenly split: composite mesh (39.0%), permanent mesh (38.0%), and biodegradable mesh (23.0%).

### Emergency clinical scenarios

**Fig. 4:**
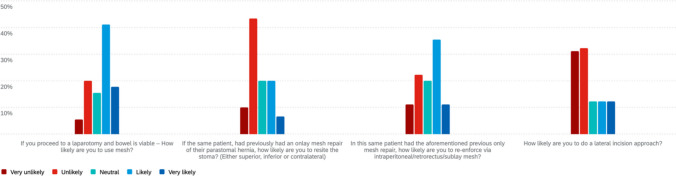
Emergency scenario of strangulated, incarcerated parastomal hernia

In the scenario of a 60-year-old female with a strangulated and incarcerated parastomal hernia post-APR (Fig. 4), most respondents (63%, 56/89) were unlikely or very unlikely to perform a lateral incision approach. When asked whether they would use mesh if laparotomy confirmed viable bowel, 52/89 (58%) reported they were likely or very likely to use mesh.

For the same patient, if a prior onlay mesh repair had been performed, 47/89 (53%) indicated they were unlikely or very unlikely to resite the stoma. When queried about the likelihood of re-reinforcement with intraperitoneal, retrorectus, or sublay mesh in this setting, 41/89 (46%) indicated they were likely or very likely to do so.

**Fig. 5:**
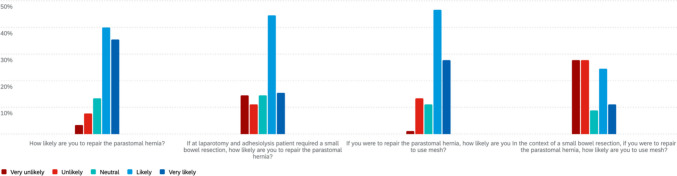
Emergency scenario of a small bowel obstruction secondary to known parastomal hernia

In the second emergency scenario involving a 58-year-old man with small bowel obstruction due to a known parastomal hernia post-APR (Fig. 5), most respondents (63%) were unlikely to repair the hernia at laparotomy if no bowel resection was required, though 25% would proceed. In this setting, 58% were likely to use mesh, 26% were unlikely, and 16% were neutral. If a small bowel resection was performed, repair remained unlikely for 53%, while 27% would proceed; 46% were likely to use mesh, 34% were unlikely, and 20% were neutral.

On subgroup analysis, two statistically significant differences were identified. First, consultant seniority was associated with stated preferences for mesh use in the emergency vignette involving small-bowel obstruction following abdominoperineal resection (21/39, 53.8% vs 10/50, 20.0%; p = 0.0015). Second, there was a strong association between preferred operative approach and mesh technique in elective repair. Surgeons favouring a minimally invasive approach predominantly selected the Sugarbaker intraperitoneal onlay technique (80.5%), whereas those opting for open access tended to use keyhole (45.8%) or retrorectus “sandwich” (37.5%) repairs (p < 0.00001).

## Discussion

This binational survey provides the most detailed snapshot to date of parastomal hernia prevention and repair practices among colorectal surgeons in Australia and New Zealand. The health systems are mixed public and private and services are geographically dispersed. We observed substantial variability from stoma creation through to elective and emergency repair, which points to a gap between evidence and day-to-day practice and a lack of shared technical standards.

Internationally, variation in stoma construction, prophylactic mesh use at index formation, and operative strategy for parastomal hernia repair appears to be the rule rather than the exception. Surveys from Europe [12] and North America[13] have reported heterogeneous approaches to fascial aperture and maturation technique, selective uptake of prophylactic mesh despite trial and guideline-level support [1], and marked variation in elective repair configuration and mesh strategy. These reports suggest that practice is shaped not only by evidence, but also by training exposure, operative volume, and local infrastructure. In Asia, guidance from the Japan Society of Coloproctology similarly describes prophylactic synthetic mesh as a preventive option at index stoma creation and supports mesh-based repair as standard for parastomal hernia, discouraging primary suture repair or stoma relocation because of high recurrence [14]. Within this broader context, our Australasian findings align with international heterogeneity, while also pointing to potential system-level contributors in geographically dispersed services, including variable access to abdominal wall expertise and differences in prosthetic governance and procurement across public and private practice.

Prophylactic mesh placement at stoma formation remains rare, reported routinely by only 12% of respondents. This aligns with international trends despite high-level evidence from trials such as ROCSS [15], PREVENT [16], and STOMAMESH [17] suggesting safety and efficacy. Concerns about infection, limited exposure to mesh techniques, and medico-legal caution may explain the reluctance [18]. Even among mesh users, there was no consensus on material type or anatomical plane, underscoring the need for procedural standardisation and practical guidance.

In Australasia, implementation barriers may be amplified by system and geography. Services are dispersed across large catchments, abdominal wall expertise may be variably available, and procurement constraints can limit consistent access to composite or biosynthetic meshes, particularly in public hospitals. Operating time, cost sensitivity, and differing local governance around prosthetic use may further discourage routine adoption even where surgeons accept the trial evidence. These factors likely contribute to the observed lack of consensus on mesh material and plane among the minority who use prophylactic mesh.

Routine stoma formation practices were similarly inconsistent. While most respondents favoured vertical fascial incisions and traditional aperture sizes (1.5–2 fingerbreadths), a notable minority opted for cruciate or larger openings. Although subtle, these variations may influence long-term outcomes such as hernia development, and their persistence highlights the lack of operative norms [19]. Consensus-driven checklists and training modules for stoma creation may help reduce this variation.

Elective repair strategies were diverse. While mesh was widely adopted, operative access (open vs minimally invasive) and mesh configuration (e.g., Sugarbaker, keyhole, retrorectus) varied markedly. Notably, open repair was associated with a greater likelihood of keyhole or retrorectus techniques, while minimally invasive access favoured Sugarbaker. This suggests that operative access often dictates technique more than clinical phenotype or evidence, reinforcing the need for comparative data to guide configuration selection.

Several additional observations merit comment. In emergency scenarios, surgeons with > 15 years’ experience were more likely to favour mesh placement, suggesting that decision-making is shaped more by seniority and comfort than consensus or guideline. Geographic disparities were also observed, with New Zealand surgeons reporting lower operative volumes than their Australian counterparts, potentially reflecting differences in centralisation or referral pathways. Overall, 70% of respondents reported fewer than five repairs annually, raising concerns about procedural proficiency and reinforcing the case for collaborative training frameworks and national audit.

Emergency decision-making exhibited the greatest heterogeneity, particularly around mesh use in contaminated fields, stoma re-siting, and redo repair strategies. These responses reflect the lack of high-quality evidence and practical guidance in reoperative scenarios. Given the high stakes and technical demands of emergency and recurrent repair, this domain represents a key target for research, protocol development, and skills training.

This study is strengthened by its structured, scenario-based design and binational scope. Several limitations warrant emphasis. First, the response rate was 25.5%. This is comparable to other surgeon surveys on parastomal hernia prevention and repair [9, 12, 13] but nonetheless introduces a material risk of non-response and selection bias. Respondents were weighted towards senior, metropolitan surgeons, and practice patterns among provincial surgeons or those with less engagement in PSH surgery may be under-represented. Second, the sampling frame was restricted to CSSANZ membership; surgeons undertaking colorectal practice outside the society were not captured, which limits external validity. Third, vignette-based responses standardise clinical context but cannot reproduce real-world constraints such as time of day, contamination, resource availability, and local rostering or supervision models. Accordingly, the observed association between seniority and emergency mesh preference should be interpreted as a difference in stated preference rather than a direct map of institutional decision-making. Finally, subgroup analyses were exploratory, with no correction for multiple comparisons.

## Conclusion

Parastomal hernia prevention and repair in Australasia is marked by wide variability in technique, material use, and clinical decision-making, with no clear consensus across elective or emergency settings. While mesh is broadly accepted in elective repair, its use in prophylaxis and contaminated fields remains inconsistent. Practice patterns appear driven more by training, experience, and institutional norms than by standardised evidence. Bridging this gap will require consensus-building, skills-based education, and formal audit through national registries. A targeted workshop or registry-based collaborative may provide the ideal platform to support technical training, research, and harmonisation of care in this complex and increasingly relevant field.

## Data Availability

De-identified survey data are available from the corresponding author on reasonable request.

## Declarations

### Reporting guidelines

This study adheres to the Checklist for Reporting Of Survey Studies (CROSS). A completed CROSS checklist is provided in Supplementary Material (Supplement 2).
